# The ‘Ride’ Feeling during Running under Field Conditions—Objectified with a Single Inertial Measurement Unit

**DOI:** 10.3390/s21155010

**Published:** 2021-07-23

**Authors:** Sabrina Bräuer, Pierre Kiesewetter, Thomas L. Milani, Christian Mitschke

**Affiliations:** Department of Human Locomotion, Chemnitz University of Technology, 09126 Chemnitz, Germany; pierre.kiesewetter@hsw.tu-chemnitz.de (P.K.); thomas.milani@hsw.tu-chemnitz.de (T.L.M.); christian.mitschke@hsw.tu-chemnitz.de (C.M.)

**Keywords:** gyroscope, ride, inertial measurement unit, biomechanics, running, midsole bending stiffness, perception

## Abstract

Foot rollover and the ‘ride’ feeling that occurs during heel–toe transition during running have been investigated mostly in laboratory settings due to the technical requirements of ‘golden standard’ measurement devices. Hence, the purpose of the current study was to investigate ‘ride’ and rollover with a heel cap-mounted inertial measurement unit (IMU) when running under field conditions to get realistic results. Twenty athletes ran on a 1 km outdoor track with five different shoe conditions, only differing in their midsole bending stiffness. The peak angular velocity (PAV) in the sagittal plane of the shoe was analyzed. The subjective evaluation of the ‘ride’ perception during heel–toe transition was rated on a visual analogue scale. The results revealed that PAV and ‘ride’ varied for the different shoes. The regression analysis showed that PAV has a significant impact on the ‘ride’ rating (R^2^ = 0.952; *p* = 0.005). The shoe with a medium midsole bending stiffness had the lowest value for PAV (845.6 deg/s) and the best rating of perceived ‘ride’ on average. Our results show that IMU can be used as a low-cost method to investigate the heel–toe transition during field-running. In addition, we found that midsole bending stiffness influenced PAV and the subjective feeling of ‘ride’.

## 1. Introduction

Several aspects play an important role when choosing a running shoe. According to a survey by Michel et al. [1], the rollover feeling is a fundamental factor for a runner to choose one shoe as “the best” from a large selection. However, there are currently only a few studies that focus on the quantification and subjective ratings of the rollover feeling during heel–toe transition [2,3,4].

Sterzing et al. [2] used an optical motion capturing system to analyze the maximum plantar flexion velocity of the foot during heel–toe transition. Their results show a relationship between the maximum plantar flexion velocity and the subjective rating of the heel–toe transition. They also show that maximum plantar flexion velocity was significantly lower for shoes with softer midsoles (*p* < 0.001).

Furthermore, using pressure-sensing insoles, Lam et al. [3] investigated the peak velocity of the center of pressure (CoP) movement in the anterior–posterior direction and the subjective rating of the heel–toe transition. They define “the feeling of the shoe during heel–toe walking or running as the foot transitions from heel to forefoot during the stance phase of the gait” as ‘ride’. Lam et al. [3] found that the shoe with a lower peak velocity for CoP movement received a significantly higher rating for ‘ride’. The results also show that some runners rated the ‘ride’ smoother in the softer shoe and some runners rated the ‘ride’ smoother in the shoe with the harder midsole.

A further study by Mally et al. [4], also using pressure-sensing insoles, investigated the influence of running velocity on ‘ride’. Their results indicated a tendency toward decreasing ‘ride’ with increasing running speed.

Optoelectronic systems (e.g., Vicon), as used by Sterzing et al. [2], have been established as the gold standard for biomechanical investigations [5,6]. However, these systems are expensive, the preparation of subjects is time-intensive, and the accuracy of the parameters can be influenced due to the low sampling rates between 50 and 500 Hz [6,7]. Furthermore, a considerable disadvantage of optoelectronic systems is that data capturing is restricted to indoor laboratory measurements [7]. Due to the limited capture space, only a small number of steps can be recorded in one overground running trial, which can influence the performance of natural movements [8]. More steps can be recorded when using optoelectronic systems for motion analysis while running on a treadmill, but differences compared to overground running have to be considered. In particular, the sagittal plane kinematics at footstrike differ between treadmill and overground running need to be considered [9].

In comparison, pressure-sensing insoles, as used by Lam et al. [3] and Mally et al. [4] (with sampling rates around 500 Hz), are an alternative to investigating biomechanical parameters during running [7]. A major advantage is that these systems can be used in the field. Nevertheless, investigations indicate problems regarding the reliability and a limited durability of pressure-sensing insoles [8]. Due to the low durability, a large number of pressure-sensing insoles are necessary to measure enough subjects, which leads to high costs.

A further option is the use of inertial measurement units (IMUs). IMUs are a cost-effective alternative to optoelectronic systems and pressure-sensing insoles [10]. They are simple to handle and, with a minimum operating range of ±32 g, they are an accurate and reliable tool to investigate human motion in the field [5,6,10,11,12,13].

However, to the best of the authors’ knowledge, no other studies have investigated rollover during heel–toe transition using IMUs. Due to the limitations of the measuring systems used in previous studies (optoelectronic systems and pressure-sensing insoles), most investigations were carried out in the laboratory over short distances or on the treadmill. Therefore, one purpose of the current study was to investigate rollover and the accompanying ‘ride’ feeling during heel–toe transition over a longer distance run on an outdoor track. The main aim of the current study was to investigate whether the ‘ride’ of a running shoe can be quantified using a single IMU. Hence, the peak angular velocity (PAV) of the foot in the sagittal plane and the subjective rating of the heel–toe transition were analyzed.

Furthermore, previous studies that investigated ‘ride’ focused on the rearfoot or forefoot midsole stiffness of the shoes. Besides these mechanical characteristics, previous studies have shown that the midsole bending stiffness (MBS) can also influence various biomechanical and physiological parameters (e.g., stride frequency and ground contact time), as well as energy cost and relative oxygen [14,15,16,17,18]. Therefore, it is becoming increasingly common to use plates (e.g., carbon plates) in the form of insoles or directly inserted into the shoe midsoles (e.g., Nike Vaporfly Next %, Adidas Adizero Pro, Hoka One One Carbon X). However, studies have shown that a specific amount of MBS is necessary for optimal performance [19]. The authors assume that most athletes perform optimally with medium MBS, while lower or higher MBS affects performance negatively. Aside from investigations regarding biomechanical and performance parameters, the influence of different MBS on ‘ride’ has not yet been investigated. However, especially during long distance runs, an unsmooth ‘ride’ can be a psychological handicap, causing athletes to run less comfortably and thus conceivably slower.

Based on previous results, it was hypothesized that:

**Hypothesis** **1** **(H1).** *The ‘ride’ of a running shoe can be quantified by the PAV by using an IMU. Lower values in PAV lead to a higher rating for ‘ride’.*

**Hypothesis** **2** **(H2).** *Differences in MBS have an impact on the PAV measured using an IMU. Medium MBS lead to significantly lower PAV.*

**Hypothesis** **3** **(H3).** *Differences in MBS have an impact on the subjective rating of ‘ride’. Medium MBS lead to significantly higher ratings for ‘ride’.*

## 2. Materials and Methods

### 2.1. Participants

Twenty healthy recreational rearfoot runners without any injuries in the last six months participated in this study (age: 29.0 ± 7.3 years, body height: 176.4 ± 4.3 cm, body mass: 69.4 ± 6.5 kg, shoe size UK 8). The participants were given information about the purpose and design of the study, and then signed an informed consent document and completed a form with their personalized data. All procedures were performed in accordance with the recommendations of the Declaration of Helsinki. This study was approved by the Ethics Committee of the Faculty of Behavioural and Social Sciences of the Chemnitz University of Technology (V-342-17-CM-Gangparameter-12062019).

### 2.2. Experimental Setup and Data Collection with Inertial Measurement Units

**Figure 1:**
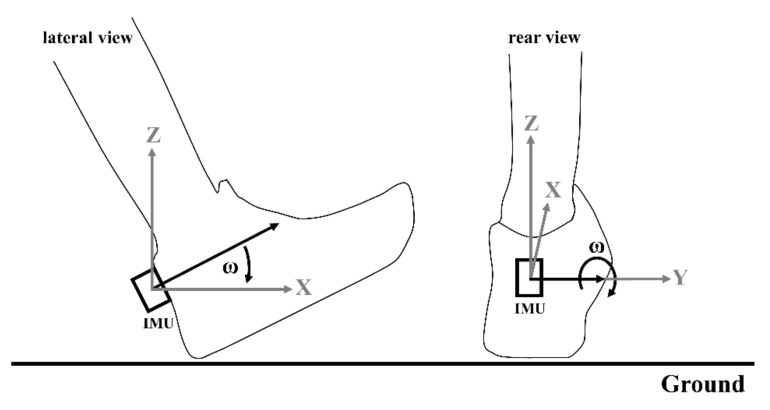
Sensor setup to measure the peak angular velocity in the sagittal plane.

To measure kinematic data and to evaluate ‘ride’, twenty subjects ran 1 km with each shoe condition on a flat asphalt outdoor track. The order of the shoes was randomized and the differences between the shoes were blinded to the participants. To measure the kinematic data, a lightweight inertial measurement unit (IMU: ICM-20601, InvenSense, San Jose, CA, USA, mass 4 g), combining a tri-axial accelerometer (measurement range ±353 m/s^2^) and a tri-axial gyroscope (measurement range ±4000 deg/s), was used. The sampling rate was set at 2000 Hz. The IMU was attached to the heel cup of the right shoe, using double-sided adhesive tape and an inelastic strap (Figure 1) [11,20]. The alignment of the IMU was parallel to the ground and to the heel cup of the shoe. The IMU was connected by a thin cable to a data logger, which recorded the data. The data logger was attached to a waist belt.

Running velocity was set to 3.0 m/s for all runners to ensure a moderate speed for all participants and to keep the fatigue effect low. To control the speed and the test procedure, the subject was accompanied by the examiner on a bicycle with a speedometer. We visually checked the rearfoot strike pattern, which was the inclusion criterion, during the individual warm ups. Subsequently, the participants completed the distance of 1 km in one pair of the shoes. When finished, they were asked to fill out a questionnaire while still wearing the shoes. On a 10 cm visual analogue scale (VAS), the participants had to rate the ‘ride’ of the shoe during the run. They were questioned: “How do you rate rollover during running?” The scale ranged from “not smooth” (0) to “very smooth” (10). After filling out the questionnaire, the participants changed to the next footwear condition and the procedure was repeated until all five pairs of shoes were tested.

### 2.3. Data Analysis

Data from the sensors were analyzed in post-processing using MATLAB R2020a (MathWorksTM, Natick, MA, USA). To separate the strides in continuous data, the accelerometer signal of the IMU (vertical axis) was 80 Hz zero-lag Butterworth high pass filtered and the first peak in the filtered signal was defined as the initial ground contact of the foot (IC) [20,21]. Furthermore, a zero-lag Butterworth low-pass filter was applied to the gyroscope data (4th order at 50 Hz) to remove noise. A previous study showed that a gyroscope can be used to precisely determine the maximum angular velocity in the frontal plane, which represents the peak eversion velocity [11]. We transferred these results to this study and assumed that the angular velocity in the sagittal plane can be used to investigate the rollover. Therefore, the maximum angular velocity in the sagittal plane of the foot was analyzed and the peak angular velocity (PAV) was extracted within 200 ms after IC. To eliminate the positive and negative acceleration at the beginning and the end of each trial, the first and last ten steps of each file were removed in the analysis.

### 2.4. Footwear Conditions

To investigate the influence of the footwear midsole material characteristics on ‘ride’, five different footwear conditions (S1–S5, men’s UK size 8, weight 269.4 ± 3.0 g) were tested in this study. The shoes were prototypes with identical outsoles, insoles, upper materials, and similar stiffness in rearfoot and forefoot areas, but different midsole bending stiffnesses as listed in Table 1. S1 was the reference footwear condition (comparable to the commercially available Puma Speed 600), whereas S2 was a more flexible shoe (flex grooves in the outsole and without a plate). According to the manufacturer’s data, the shoes S3, S4, and S5 were provided with a stiffer plate compared to the reference shoe S1. S2 was the most flexible (63.0 N) and S5 (98.0 N) was the least flexible shoe condition. S3 had a medium MBS (76.0 N).

### 2.5. Statistical Analysis

Mean and standard deviations (mean ± SD) were calculated for the biomechanical and subjective variables. Given that variables were normally distributed according to the Shapiro–Wilk test, a one-way analysis of variance (ANOVA) for repeated measurements followed by the Bonferroni post-hoc test was used to determine whether differences existed between the footwear conditions regarding PAV and the subjective variable. Statistical significance was set at α = 0.05 for all analyses. In addition, effect size (Cohen’s d) was calculated to quantify the magnitude of differences if statistical significance was found. The coefficients were interpreted as trivial (d < 0.2), small (d < 0.5), medium (d < 0.8), or large effects (d ≥ 0.8) [22]. Additionally, a linear regression analysis was carried out to investigate the relationship between PAV and ‘ride’. This regression analysis was based on the calculation of the median over the 20 participants for PAV and ‘ride’.

## 3. Results

### 3.1. Biomechanical Test: Peak Angular Velocity

**Figure 2:**
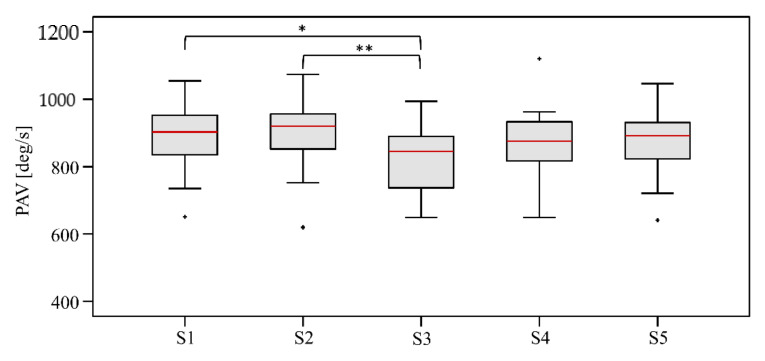
Comparison of peak angular velocity (PAV) between shoe conditions (* *p* < 0.05; ** *p* < 0.01).

In mean, 451.2 ± 20.2 strides were analyzed for all shoe conditions. The analysis of PAV revealed the lowest value for S3, with a median of 845.6 deg/s (648.9–993.7 deg/s), and the highest value for S2, with a median of 919.5 deg/s (619.7–1073.1 deg/s). The ANOVA showed a significant main effect (*p* < 0.001) for PAV between the five different shoe conditions. The Bonferroni post-hoc test revealed a significant difference between S1 and S3 (*p* = 0.014), and a highly significant difference between S2 and S3 (*p* = 0.008) (Figure 2). Cohen’s d showed large effects (d ≥ 0.8) for both significant pairwise comparisons.

**Figure 3:**
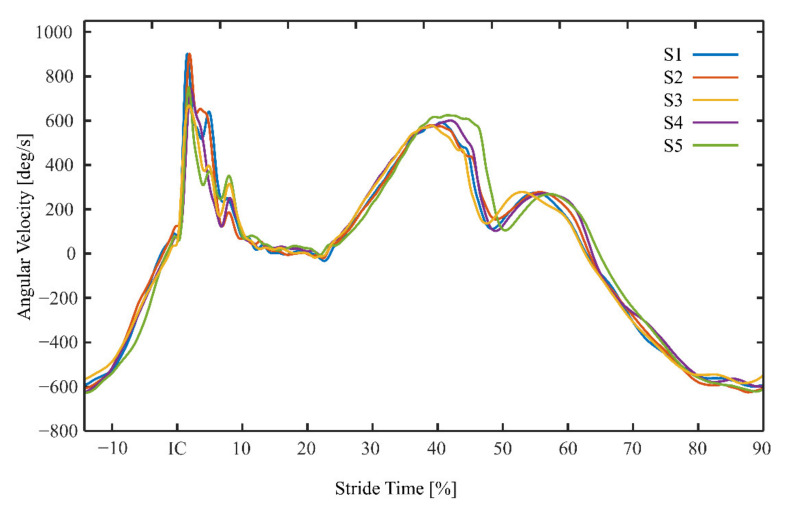
Exemplary curve of the angular velocity for one subject during one stride cycle. Determination of peak angular velocity within the first 20% of stride cycle time after initial ground contact of the foot (IC).

As shown in the example for one subject in Figure 3, the lowest PAV was found in S3.

### 3.2. Subjective Results: ‘Ride’

**Figure 4:**
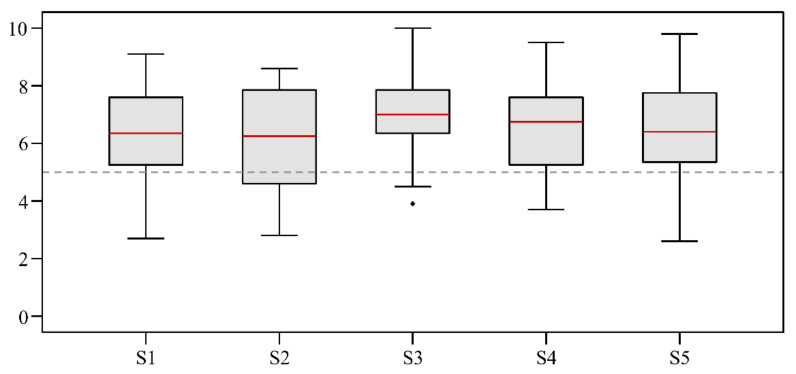
Comparison rating ‘ride’ between shoe conditions (“not smooth” (0) to “very smooth” (10)).

The subjective evaluation for ‘ride’ showed the best rating for S3, with a median of 7.00 (3.90–10.00), and the lowest rating for S2, with a median of 6.25 (2.80–8.60). The ANOVA revealed no significant mean effect (*p* = 0.263) between the ratings for ‘ride’ (Figure 4).

### 3.3. Correlation Peak Angular Velocity and ‘Ride’

**Figure 5:**
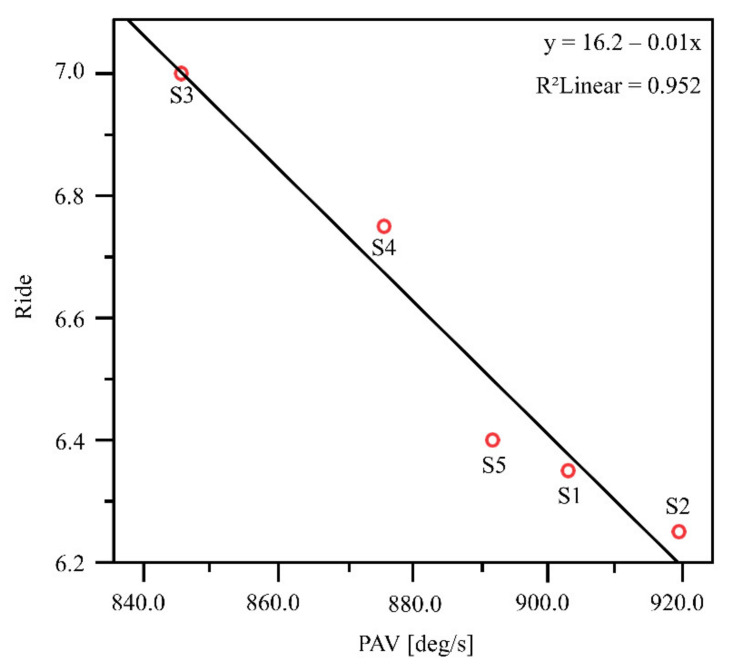
Correlation between PAV and ‘ride’. Analysis was based on the calculation of the median over the 20 participants for PAV and ‘ride’ for each of the five shoe conditions.

The regression analysis revealed a significant interaction between PAV and ‘ride’ (F = 59.479; *p* = 0.005). Thus, PAV seems to have an impact on ‘ride’. As shown in Figure 5, a negative correlation (r = −0.976) was found between PAV and ‘ride’. The effect size showed a strong effect (f = 4.45).

## 4. Discussion

The main aim of the current study was to investigate whether the ‘ride’ of a running shoe can be quantified with a single IMU, which is attached at the heel cap of the shoe. The runs were performed under field conditions to get a realistic rating of the runners from the heel-toe transition. It was hypothesized (H1) that lower values in PAV, measured with an IMU, lead to a higher subjective evaluation of ‘ride’, and that there would be a high correlation between both of these parameters. Furthermore, it was hypothesized that MBS of shoes can influence running style, which leads to lower PAV (H2) and higher (respectively better) ‘ride’ ratings (H3) for shoes with medium MBS.

H1 was confirmed: The analysis of the PAV revealed the lowest value for S3, with a median of 845.6 deg/s, and the highest value for S2, with a median of 919.5 deg/s. Significant differences were found between S1 and S3 and between S2 and S3 with large effects (d ≥ 0.8). The analysis of the subjective evaluation of ‘ride’ revealed no significant mean effects, but the shoe S3 received the highest ratings (median: 7.00) and shoe S2 received the lowest ratings (median: 6.25). This means that the shoe with the lowest PAV during running (S3) was rated best regarding ‘ride’. In contrast, the shoe S2, which led to the highest PAV, was rated worst regarding ‘ride’. The regression analysis confirmed the interaction between PAV and ‘ride’ over all five shoe conditions with a high coefficient of R^2^ = 0.952. Thus, PAV seems to have a significant impact on ‘ride’. Lower values in PAV during running led to higher subjective ratings of ‘ride’. Previous studies also found a relationship between the peak velocity of plantar CoP and ‘ride’ [3], and between the maximum plantar flexion velocity and the feeling during rollover [2]. Thus, our results correspond with the results of Sterzing et al. [2] and Lam et al. [3]. The agreement between our findings and previous investigations [2,3] shows that using IMUs attached at the heel cap of the shoe provide a relatively low-cost but reliable method to investigate the heel–toe transition during running and to objectify the ‘ride’ feeling of the runner. However, it should be considered that the measured angular velocity at the heel cup of the shoe in the sagittal plane represents the rollover behavior of the shoe and not of the foot directly. In some cases (e.g., when sliding inside the shoe), it is possible that the coordinate axes of foot and shoe do not match precisely.

H2 was also confirmed: Our results show that the MBS of the shoe can influence running style. The lowest PAV was found for the shoe with medium MBS (S3). Running in shoes with lower (S1 and S2) or higher (S4 and S5) MBS resulted in higher PAV values. It seems that the runner adapts the heel–toe transition due to the altered MBS. In contrast to the studies of Lam et al. [3] and Sterzing et al. [2], the effect of the material characteristics in the rearfoot and forefoot areas on running style can be excluded in our study due to the similar properties in these areas for all shoes.

The angular velocities in the current study (845.6–919.5 deg/s) were slightly lower for all shoe conditions than those found by Sterzing et al. [2] (peak plantarflexion velocity between 913.4–991.8 deg/s). This could be due to the differences in running speeds implemented in the studies. Orendurff et al. [23] found that a higher running speed leads to significantly higher ankle angles in the sagittal plane. Thus, with a higher running speed, higher angular velocities are expected. The running speed in the current study was set at 3.0 m/s and Sterzing et al. [2] had a running speed of 3.3 m/s.

H3 had to be rejected: The analyses of the ‘ride’ rating found that MBS has an impact on ‘ride’; however, the differences were not significant. The medium MBS (S3) tended to receive a higher or better rating for ‘ride’ than for shoes with lower (S1 and S2) or higher MBS (S4 and S5). Sterzing et al. [2] found significant differences (*p* < 0.001) in the subjective rating of the heel–toe transition. However, the effect size of their findings was low (f = 0.193), and they tested shoes with different material characteristics in the rearfoot and forefoot areas. Lam et al. [3] analyzed shoes with different rearfoot properties and had mixed results for ‘ride’ ratings; sometimes the higher ratings were found for the compliant shoe and sometimes in the harder shoe. They suspected that individual factors (such as anthropometric properties) could also have an impact. However, there is no other investigation that has evaluated the influence of different MBS on ‘ride’. Since the rearfoot and forefoot properties for the shoes used in the current study were the same, our results found that MBS also has a relevant impact on ‘ride’. A certain MBS seems to be necessary for a smoother ‘ride’. Our investigation found that MBS values that were too low or too high led to a ‘ride’ that was less smooth. Our finding that medium MBS led to the optimal ‘ride’ complements the results from the study by Roy and Stefanyshyn [19]. Their findings showed that most athletes performed optimally with a medium MBS, while performance dropped with lower or higher MBS.

This study has a few limitations that should be mentioned. First, only the ‘ride’ for rearfoot strike running was investigated. Further studies should also investigate ‘ride’ for midfoot and forefoot strike running. A second limitation is that running velocity was set to 3.0 m/s for all runners to ensure a moderate speed for all participants and to make the results comparable regarding PAV. However, the given speed could influence the running style, because some runners’ usual pace might be slower or faster than 3.0 m/s. Further studies could investigate the difference in ‘ride’ ratings when running at a given and at an individually-selected running velocity. The third limitation is that the measurements with the IMUs were only on the right side. Additional studies could investigate both sides to compare them, as was done in the study by Lam et al. [3]. Furthermore, runners had to rate ‘ride’ after 1 km of running with the respective shoe condition. Follow-up studies could investigate the ‘ride’ feeling after a longer running distance (for example, 5 km). To measure longer distances, the number of shoes in the test set-up should be reduced.

## 5. Conclusions

Our study found that ‘ride’ can be objectified by measuring PAV with an IMU. It seems that PAV has a significant impact on ‘ride’. A runner’s perception and evaluation of the heel–toe transition might be a reliable parameter to determine whether a runner has a high or low PAV in a running shoe. Lower values for PAV seem to lead to a smoother perceived ‘ride’. Furthermore, MBS also seems to have an impact on ‘ride’. A medium MBS led to the smoothest ‘ride’. The values for PAV showed that the shoe with medium MBS was the best condition for low PAV. The shoe conditions in our study only differed regarding MBS. Additional research could investigate medium MBS combined with differences in forefoot and rearfoot stiffness to further improve ‘ride’. Furthermore, our results revealed a large distribution of values around the median. This could be the result of the individual running style (PAV) as well as the individual perception of ‘ride’. In follow-up studies, the aim could be to investigate reasons for the variation within the same footwear condition and between the different footwear conditions.

## Figures and Tables

**Table 1:** Shoes used in this study with different midsole bending stiffnesses. All shoes were men’s size 8 Puma shoes. Required force to bend the shoe in the forefoot area 45 degree.

Shoe	S1	S2	S3	S4	S5
Midsole bending stiffness (N)	71.5	63.0	76.0	84.0	98.0

## Data Availability

The data presented in this study are available on request from the corresponding author.
